# Identification of circulating biomarkers in sera of *Plasmodium knowlesi*-infected malaria patients – comparison against *Plasmodium vivax* infection

**DOI:** 10.1186/s12879-015-0786-2

**Published:** 2015-02-06

**Authors:** Yeng Chen, Choon K Chan, Jesinda P Kerishnan, Yee L Lau, Yin-Ling Wong, Subash CB Gopinath

**Affiliations:** Department of Oral Biology & Biomedical Sciences, Faculty of Dentistry, University of Malaya, 50603 Kuala Lumpur, Malaysia; Oral Cancer Research and Coordinating Center, Faculty of Dentistry, University of Malaya, Kuala Lumpur, Malaysia; Institute for Research in Molecular Medicine, Universiti Sains Malaysia, Gelugor, Penang Malaysia; Department of Parasitology, Faculty of Medicine, University of Malaya, Kuala Lumpur, Malaysia; Institute of Nano Electronic Engineering (INEE), & School of Bioprocess Engineering, Universiti Malaysia Perlis, Kangar, Perlis Malaysia

**Keywords:** *Plasmodium knowlesi*, *Plasmodium vivax*, Malaria, Immunoproteomics, Antigenicity

## Abstract

**Background:**

*Plasmodium knowlesi* was identified as the fifth major malaria parasite in humans. It presents severe clinical symptoms and leads to mortality as a result of hyperparasitemia in a short period of time. This study aimed to improve the current understanding of *P. knowlesi* and identify potential biomarkers for knowlesi malaria.

**Methods:**

In the present study, we have employed two-dimensional gel electrophoresis-coupled immunoblotting techniques and mass spectrometry to identify novel circulating markers in sera from *P. knowlesi*-infected patients. Specifically, we have compared serum protein profiles from *P. knowlesi*-infected patients against those of healthy or *P. vivax*-infected individuals.

**Results:**

We identified several immunoreactive proteins in malarial-infected subjects, including alpha-2-HS glycoprotein (AHSG), serotransferrin (TF), complement C3c (C3), hemopexin (HPX), zinc-2-alpha glycoprotein (ZAG1), apolipoprotein A1 (Apo-A1), haptoglobin (HAP), and alpha-1-B-glycoprotein (A1BG). However, only TF and HPX displayed enhanced antigenicity and specificity, suggesting that they might represent valid markers for detecting *P. knowlesi* infection. Additionally, six *P. knowlesi*-specific antigens were identified (K15, K16, K28, K29, K30, and K38). Moreover, although HAP antigenicity was observed during *P. vivax* infection, it was undetectable in *P. knowlesi*-infected subjects.

**Conclusions:**

We have demonstrated the application of immunoproteomics approach to identify potential candidate biomarkers for knowlesi malaria infection.

## Background

Malaria has been known to be caused by major human malaria parasites – *Plasmodium falciparum*, *P. vivax*, *P. malariae* and *P. ovale. P. knowlesi* which was initially found in only long-tailed (*Macaca fascicularis*) and pig-tailed (*Macaca nemestrina*) macaques [1]. It has been recognized as the fifth species of Plasmodium that causes malaria infection in humans [2,3]. Several cases of *P. knowlesi* infection in humans were reported in Malaysian Borneo, Myanmar, Philippines, Singapore, and Thailand [1,4], supporting the notion that *P. knowlesi* represents a major cause of malaria in Southeast Asia. *P. knowlesi* is the only malaria species that has a 24-hour asexual reproduction cycle (quotidian). Therefore, patients infected with *P. knowlesi* can easily reach lethal parasite densities in a relatively short period of time [5]. Respiratory distress, renal dysfunction, jaundice, hypoglycaemia and severe anaemia are the general clinical manifestations of severe malaria infection [6]. In addition, the severity of knowlesi malaria was found to be associated with hyperparasitemia and this has been reported in Malaysia and in other Southeast Asia regions [5,6].

Like other malaria species, identification of *P. knowlesi* infection is achieved through examination of thick and thin blood films followed by Giemsa microscopy. This method uses specific morphological characteristics to differentiate parasites. However, accurate diagnosis of *P. knowlesi* by microscopy is often limited by the fact that *P. knowlesi* bares strong morphological resemblance *to P. falciparum* (early trophozoite stage) and *P. malariae* (erythrocytic stages) [7]. Thus, diagnosis of *P. knowlesi* usually requires molecular detection methods, which are performed in reference laboratories. In this regard, polymerase chain reaction (PCR) and molecular characterization currently represent the most reliable detection methods for *P. knowlesi* infection. Nevertheless, PCR-based techniques are not suitable for routine identification, since this method requires parasite DNA and is time consuming. In addition, *P. knowlesi* is frequently misdiagnosed as *P. malariae* through PCR and conventional microscopy [5]. Currently, there are no commercially available malaria rapid diagnostic tests are designed specifically for *P. knowlesi* detection. Although Plasmodium lactate dehydrogenase (pLDH) assay is more reliable in detecting *P. knowlesi* infection, the cross-reactivity of *P. knowlesi* with *P. falciparum*-specific and *P. vivax*-specific pLDH has been shown [8]. Therefore, as stated by Cox-Singh et al. [5], there is currently a fundamental need for effective and practical diagnostic methods, which will not only contribute to reduced malaria-associated complications and mortality, but also facilitate global malaria control.

Notably, host biomarkers can be used to assess the risk of infection, examine protection against active diseases, or to determine therapeutic responses. Indeed, employing gene profiling, Schaecher et al. [9] has identified host biomarkers that can differentiate between lethal and non-lethal blood stages of murine malaria. Serum samples collected during the course of infection were utilized to analyze differential protein expression patterns, which were found to correlate with the degree of infection. Such data can provide insight into the cell regulatory mechanisms that participate in pathogenesis, immune responses and host recovery. Moreover, the identified proteins could be measured by serological tests to detect variants of malaria parasites while conducting epidemiological studies or implementing control programs. For example, serum angioprotein I and angioprotein 2/L have been suggested as diagnostic and prognostic biomarkers as well as potential therapeutic targets in cerebral malaria [10]. In the present study, we have examined the proteomic profiles of serum from *P. knowlesi*-infected patients to identify distinctive immunological protein features. Specifically, we have compared serum protein profiles from *P. knowlesi*-infected patients against those of healthy or *P. vivax*-infected subjects. Our findings have the potential to enhance our understanding of *P. knowlesi* and might contribute to the development of novel diagnostic approaches.

## Methods

### Clinical samples

From 200 serum samples previously screened for malaria parasites [11], we selected 15 samples for the current study. Notably, these samples corresponded to patients who were newly diagnosed with either *P. knowlesi* (n = 9, parasitemia range: 0.04-22.80%, age range: 29–55 years, Male, Malaysian) or *P. vivax* (n = 6, parasitemia range: 0.10-0.50%, age range: 29–50 years, Male, non-Malaysian Asian). Additionally, 23 serum samples were collected from normal healthy individuals and were used as a control group (age range: 29–50 years, Male, Malaysian). All samples were obtained with patients’ written consent, and this study was approved by the University of Malaya Medical Centre Ethical Committee in accordance with ICH–GCP guidelines for good clinical practice and the Declaration of Helsinki (PPUM/MDU/300/04/03).

### Two-dimensional electrophoresis (2-DE)

Two-dimensional electrophoresis (2-DE) was performed as previously described by Chen et al. [12]. Briefly, 10 μl of unfractionated whole human serum (either individual serum or pooled sera) was subjected to isoelectric focusing using 13-cm rehydrated precast immobilized dry strips (pH 4–7) (GE Healthcare Bio-Sciences, Uppsala, Sweden). For the second dimension, focused sample within the strips was subjected to electrophoresis using an 8–18% gradient polyacrylamide gel in the presence of sodium dodecyl sulphate (SDS). All samples were analyzed in duplicate. The 2-DE gels were silver stained as described by Heukeshoven and Dernick [13]. For mass spectrometric analysis, gels were stained using Coomassie Brilliant Blue (CBB) or a modified mass spectrometry (MS) silver staining method, as described by Shenvchenko et al. [14]. CBB gel plugs were used instead of silver-stained gel plugs when a higher peptide concentration was expected from in-gel digestion.

### Mass spectrometry analysis and database search

Selected spots were excised and subjected to in-gel tryptic digestion using the commercially available ProteoExtract™ All-in-One Trypsin Digestion Kit (Calbiochem, Darmstadt, Germany). Mass spectrometry (MS) analysis was performed at the Faculty of Biological Sciences Proteomic Centre, National University of Singapore. After digestion, the resulting peptides were mixed with CHCA matrix solution (5 mg/ml of cyano-4-hydroxy-cinamic acid in 0.1% trifluoroacetic acid [TFA] and 50% acetonitrile [ACN]) in 1:2 ratio and spotted onto a matrix-assisted laser desorption/ionization (MALDI) target plate. Peptide mass spectra were obtained using an ABI 4800 Proteomics Analyzer MALDI-TOF/TOF Mass Spectrometer (Applied Biosystems, Foster City, CA, USA). For MS analysis, 1,000 shots were accumulated for each sample. MS data were automatically obtained with the five most intense ions for MS/MS. Peptides were subsequently subjected to MS/MS analyses using air with collision energy of 2 kV and a collision gas pressure of ~1×10^−6^ Torr. The stop conditions were set to accumulate approximately 2,000 to 3,000 shots, depending on the quality of the spectra. The Mascot search engine (version 2.1; Matrix Science, London, UK) was used to analyze all of the tandem MS results. Also, GPS Explorer™ software (version 3.6; Applied Biosystems) was employed in combination with the Mascot search engine for peptide identification. The search parameters allowed for N-terminal acetylation, C-terminal cysteine carbamidomethylation (fixed modification), and methionine oxidation (variable modification). The peptide and fragment mass tolerance were set to 100 ppm and ±0.2 Da, respectively. Moreover, peptide mass fingerprinting (PMF) parameters for the data search were as follows: one missed cleavage allowed in trypsin digest; monoisotropic mass value; ±0.1 Da peptide mass tolerance; and 1+ peptide charge state. Initial protein identification was determined by comparing peptide masses to a database of tryptic peptides from known proteins (ProteinPilot proteomics software [4800] Proteomic Analyzer; Applied Biosystems), and a score was assigned based on similarity to theoretically and experimentally determined masses. Analyses were conducted using International Protein Index (http://www.ebi.ac.uk/IPI/), NCBI, Unigene (version 3.38), and PlasmoDB (version 8.0; http://plasmodb.org/plasmo/) databases for human proteomics. There were a total of 10,719 entries included in the database search. Search scores of >82 (Mascot NCBI database) or >30 (Mascot search engine using PlasmoDB) were considered as significant.

### Immunoblotting

For immunoblotting, we analyzed 10 μl of pooled, unfractionated human serum. Following electrophoresis, the 2-DE gels for the pooled serum samples were grouped into five categories: (a) normal pooled sera probed with normal pooled sera, (b) normal pooled sera probed with *P. knowlesi* pooled sera, (c) *P. knowlesi* pooled sera probed with normal pooled sera, (d) *P. knowlesi* pooled sera probed with *P. knowlesi* pooled sera, (e) *P. knowlesi* pooled sera probed with *P. vivax* pooled sera. Each of the gels was transferred onto nitrocellulose membrane using the Multiphor II Novablot semi-dry system (GE Healthcare, Sweden). The blotted nitrocellulose membranes were then blocked with SuperBlock (Pierce, USA) and washed three times with Tris-buffered saline–Tween 20 (TBST). The membranes were subsequently incubated overnight (4°C) with the indicated primary antibodies, which corresponded to *P. knowlesi* infection, *P. vivax* infection, or normal healthy controls (all diluted at 1:50). After another washing step, the membranes were incubated with monoclonal anti-human Immunoglobulin M (IgM) conjugated to horseradish peroxidase (HRP) (Invitrogen, USA) at a dilution of 1:6,000 for 1 h at room temperature. The resulting immunocomplexes were visualized using chemiluminescent blotting reagent (Pierce, USA) and X-ray film (18 × 24 cm; Kodak).

### Differential image and data analysis

We utilized LabScan image scanner (version 5; Amersham, Germany) to capture and store the 2-DE gel images. PD-Quest™ 2-D gel analysis software (Version 8.0.1, Bio-Rad) was used to detect, match, and quantify the distinct protein spots. In order to identify differentially expressed serum proteins, the percentages of volume contributions (vol%) were calculated (i.e., the spot volume of a specific protein as a percentage of the total spot volume of all proteins in the gel, including unresolved peptides).

### Statistical analysis

Protein levels in the gels are presented as mean vol%  ± SD (standard deviation). The variance ratio test (F) was used to analyse differences between control subjects and patients. Correlations between the variables were examined using GraphPad Prism 5 software. A p-value of less than 0.05 was considered as statistically significant.

### Functional enrichment and protein interaction analysis

Functional enrichment and protein interactions were analysed using web-based bioinformatics tools. DAVID v6.7 (Database for Annotation, Visualization and Integrated Discovery) was employed for protein functional enrichment analysis [15,16]. DAVID bioinformatics provide a comprehensive biological knowledgebase of functional annotation tools for understanding the biological meaning behind large lists of genes or proteins. The functional categorization is considered significant when the p-value is less than 0.05. The identified host-specific proteins were further evaluated using STRING v9.1 (Search Tool for the Retrieval of Interacting Genes), which is an application that aggregates available databases of known and predicted protein–protein associations [17].

## Results

### Differential expression of antigenic proteins

**Figure 1:**
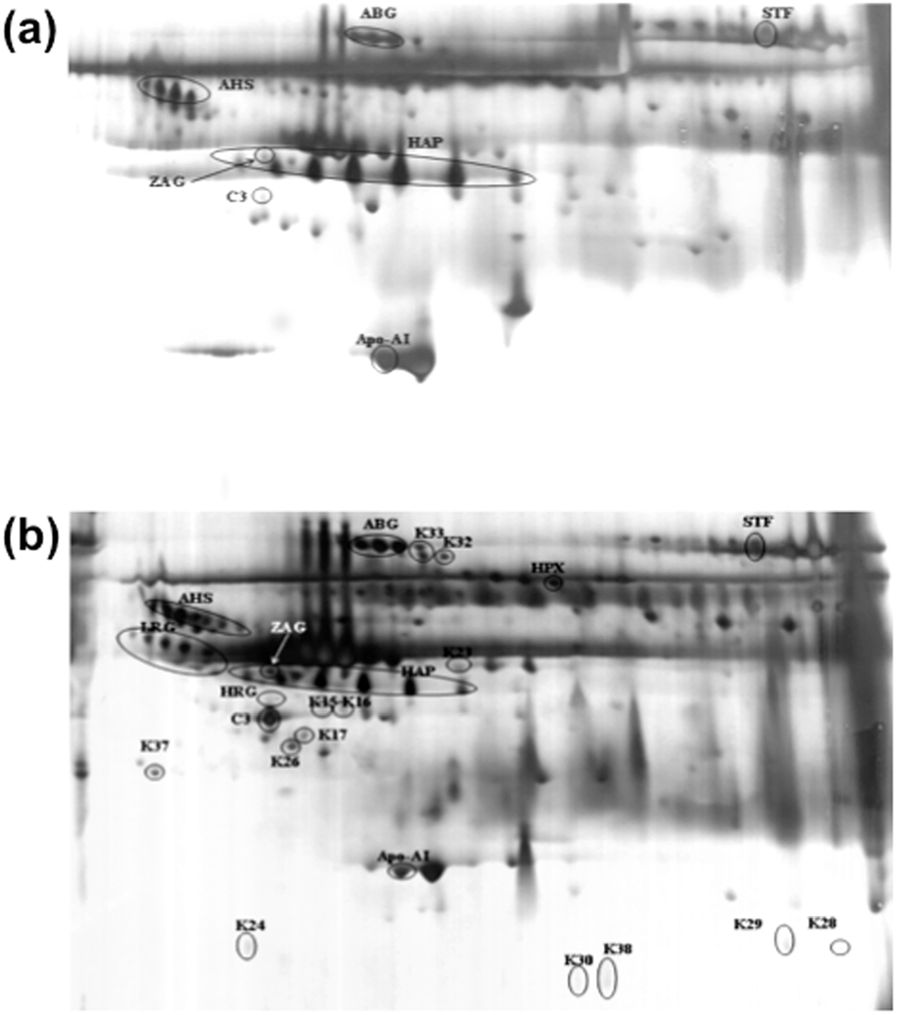
**Representative 2-DE serum protein profiles for (a) control (b)** ***P. knowlesi*** **-infected individuals.** Unfractionated serum samples from patients and controls were subjected to 2-DE and silver stained. Protein spots were compared and analysed using PD-Quest^™^ 2-D gel analysis software (version 8.0.1, Bio-Rad).

We have performed a 2-DE assay that allowed for rapid detection and differentiation of *P. knowlesi* from other *Plasmodium* species. We first separated unfractionated normal control sera samples with 2-DE and observed high-resolution profiles, which were made up of several distinct protein clusters (Figure 1a). Sera samples from *P. knowlesi*-infected patients, presumably containing *P. knowlesi* antigens, were also separated by 2-DE (Figure 1b). Subsequently, the 2-DE profiles from these patients were compared with those obtained from normal individuals, revealing key differences in the expression of several serum proteins. Notably, knowlesi malaria patients displayed protein spots/clusters that appeared to undergo up- or down-regulation (Figure 1b).

### Identification of expressed biomarkers by mass spectrometry

We analysed the protein clusters using PD-Quest TM 2-DE gel analysis software (version 8.0.1, Bio-Rad), which indicated that there were a total of 200 and 124 protein spots detected by 2-DE for the *P. knowlesi* and normal samples, respectively. Therefore, 76 protein spots were differentially observed in malaria knowlesi sera compared to normal controls.

We further identified the following differentially-expressed host-specific proteins by MS: alpha-2-HS glycoprotein (AHSG), serotransferrin (TF), complement C3c (C3), hemopexin (HPX), zinc-2-alpha glycoprotein (ZAG1), apolipoprotein A1 (Apo-A1), haptoglobin (HAP), and alpha-1-B-glycoprotein (A1BG). All of the proteins were aberrantly expressed in patient samples as compared to normal controls. Indeed, as shown in the Table 1, we observed significant increases in C3, ZAG1, HPX, AHS, and TF during knowlesi malaria infection (fold up-regulation: 8.99, 3.40, 1.60, 1.25, and 1.15, respectively; p < 0.05). On the other hand, the expression of HAP, Apo-A1, and A1BG were significantly decreased (fold down-regulation: 0.42, 0.34, and 0.53; p < 0.05) in knowlesi samples (Figure 1a-b). Additionally, we detected the following malaria-specific antigens: K15, K16, K17, K23, K26, K28, K29, K30, K32 K33, and K38 (Tables 1, 2, and 3).

**Table 1:** **The relative expression of host specific proteins among the sera of malaria patients**

**Host specific proteins**	**Fold changes**
1. Alpha-2-HS glycoprotein (AHSG)	1.28
2. Serotransfererrin (TF)	1.15
3. Complement C3c (C3)	8.99
4. Hemopexin (HPX)	1.60
5. Zinc-2-alpha glycoprotein (ZAG1)	3.40
6. Haptoglobin (HAP)	0.42
7. Apolipoprotein A-I (Apo-A1)	0.34
8. Alpha-1-B-glycoprotein (A1BG)	0.53

Fold change measures the degree of change in the protein of the *P. knowlesi*-infected individuals (n = 9), compared to normal controls (n *= 2*3). This is measured by dividing the average spot intensity in the infected persons by the average spot intensity in the controls.

**Table 2:** **Host antigenic proteins show on the 2-DE immunoblotted nitrocellulose membrane**

**Host antigenic proteins**	**Category**				
	**(a)**	**(b)**	**(c)**	**(d)**	**(e)**
1. AHSG	-	/	-	/	/
2. TF	-	-	-	/	-
3. C3	-	-	-	/	/
4. HPX	-	-	-	/	-
5. ZAG1	-	-	-	/	/
6. HAP	-	-	-	-	/
7. Apo-A1	-	/	-	/	/
8. A1BG	-	/	-	/	/
9. HRG	-	-	-	-	/

/Proteins of the patients or normal pooled serum recognized by the primary antibody.

-Proteins of the patients or normal pooled serum not recognized by the primary antibody.

**Table 3:** **Antigenic proteins show on the 2-DE immunoblotted nitrocellulose membrane**

**Antigenic proteins**	**Category**				
	**(a)**	**(b)**	**(c)**	**(d)**	**(e)**
1. K15	-	-	-	(/)	-
2. K16	-	-	-	(/)	-
3. K17	-	-	-	-	/
4. K23	-	-	-	-	/
5. K26	-	-	-	-	/
6. K28	-	-	-	(/)	-
7. K29	-	-	-	(/)	-
8. K30	-	-	-	(/)	-
9. K32	-	-	-	/	/
10. K33	-	-	-	/	/
11. K38	-	-	-	(/)	-

(/)Antigens specifically detected in the knowlesi serum.

/Proteins of the patients or normal pooled serum recognized by the primary antibody.

-Proteins of the patients or normal pooled serum not recognized by the primary antibody.

The above results were confirmed through the use of MALDI-TOF/TOF and database searches. The PlasmoDB database was employed to blast MS/MS-identified peptides against the hypothetical *P. knowlesi* H strain proteome, which is only comprised of computationally predicted sequences [18]. This database contains the genomes of various mammalian Plasmodium species (*P. falciparum, P. knowlesi, P. vivax, and P. yoelii*), which range from 23 to 27 Mb across 14 chromosomes and comprise approximately 5,500 genes (http://plasmodb.org/plasmo/). A remarkable 77% of these genes represent orthologous between these four species, and almost one-half of the genes encode conserved hypothetical proteins of unknown function. However, although PlasmoDB contains the most up-to-date annotation on *P. knowlesi* sequences, it is incomplete. Thus, it cannot be ruled out that some genes may be missing or incorrectly annotated in the PlasmoDB database. For this reason, experimental verification will be needed to assess whether current data offer a comprehensive view of the *Plasmodium* proteome, especially in the case of *P. knowlesi*. Nevertheless, our findings provide evidence to support the existence of some of these predicted sequences. The Mascot accession numbers, isoelectric points (pI), and molecular mass (Mr) values associated with the identified proteins are listed in Tables 4 and 5.

**Table 4:** **Mass spectrometric identification of host-specific protein spot clusters from serum protein profiles using MASCOT search engine and NCBI database**

**Spot ID**	**Mascot accession number**	**pI**	**Theoretical mass (Da)/pI**	**Sequence coverage**	**Search score**	**Queries match**	**Expected value**
1. Chain C, Human Complement Component C3c (C3)	gi|78101271	4.79	40217	10%	218	6	1.8e^−015^
2. Transferrin (TF)	gi|553788	6.00	55233	13%	82	10	0.082
3. Chain A, Crystal Structure Of Lipid-Free Human Apolipoprotein A-I (ApoA1)	gi|90108664	5.27	28061	62%	357	26	2.3e^−029^
4. Leucine-rich α_2_-glycoprotein precursor [Homo sapiens] (LRG1)	gi|16418467	6.45	38382	31%	358	16	1.8e^−029^
5. Hemopexin, isoform CRA_d [Homo sapiens] (HPX)	gi|119589127	6.24	43781	13%	112	8	7.3e^−005^
6. α_1_-B-glycoprotein (A1BG)	gi|69990	5.65	52479	39%	449	20	1.5e^−038^
7. α_2_-HS-glycoprotein, isoform CRA_b [Homo sapiens] (AHSG)	gi|119598594	4.81	23148	6%	121	2	9.2e^−006^
8. Haptoglobin [Homo sapiens] (HAP)	gi|3337390	6.14	38722	28%	202	15	7.3e^−014^
9. Histidine-rich glycoprotein precursor [Homo sapiens] (HRG)	gi|4504489	7.09	60510	13%	114	10	4.6e^−005^
10. Chain B, Human Zinc-α_2_-glycoprotein (ZAG1)	gi|4699583	5.70	31854	51%	523	22	5.8e^−046^
11. Fibrinogen gamma chain (FGG)	gi|930064	6.49	24337	45%	56	10	33

**Table 5:** **Mass spectrometric identification of unknown protein spot clusters from serum protein profiles using MASCOT search engine, NCBI database and Plasmo DB**

**Spot ID**	**Mascot accession number**	**ID**	**pI**	**Theoretical mass (Da)/pI**	**Sequence coverage**	**Search score**	**Queries match**	**Expected value**
		**Number**						
Plasmodium_knowlesi_strain_H | product = hypothetical protein, conserved in Apicomplexan s	psu|PKH_030860	K15	8.42	57570	16%	37	8	1.1
Plasmodium_knowlesi_strain_H | product = conserved Plasmodium protein, unknown function	psu|PKH_050250	K16	9.20	52407	1%	26	2	13
Plasmodium_knowlesi_strain_H | product = dynein light chain 1, putative | location = Pk_stra	psu|PKH_050850	K17	8.75	23061	34%	34	7	2.1
Plasmodium_knowlesi_strain_H | product = conserved Plasmodium protein, unknown function |	psu|PKH_093010	K23	5.08	32237	34%	46	10	0.13
Plasmodium_knowlesi_strain_H | product = translation intiation factor IF-2 | location = Pk_s	psu|PKH_050380	K24	8.77	137165	5%	28	12	7.7
Plasmodium_knowlesi_strain_H | product = conserved Plasmodium protein	PKH_050250	K26	9.20	52407	1%	29	2	6.9
Plasmodium_knowlesi_strain_H | product = conserved Plasmodium protein, unknown function	psu|PKH_091010	K28	8.32	20628	8%	38	3	0.82
Plasmodium_knowlesi_strain_H | product = conserved Plasmodium protein, unknown function	psu|PKH_050250	K29	9.20	52407	1%	15	2	1.6e^+002^
Plasmodium_knowlesi_strain_H | product = conserved Plasmodium protein, unknown function	psu|PKH_091010	K30	8.32	20628	8%	32	3	8.32
Plasmodium_knowlesi_strain_H | product = SOH1 homologue, putative | location = Pk_strainH_ch	psu|PKH_126910	K32	6.90	16303	20%	42	9	0.37
Plasmodium_knowlesi_strain_H | product = hypothetical protein, conserved in Apicomplexan s	psu|PKH_031640	K33	9.43	9570	24%	25	3	18
Plasmodium_knowlesi_strain_H | product = dihydrolipoamide dehydrogenase, putative | ocate	psu|PKH_051660	K37	9.21	74131	1%	16	2	1.2e^+002^
Plasmodium_knowlesi_strain_H | product = conserved Plasmodium protein, unknown function	psu|PKH_050250	K38	9.20	52407	1%	31	2	4.4

### Detection of immunocomplexed biomarkers by 2-DE immunoblotting

**Figure 2:**
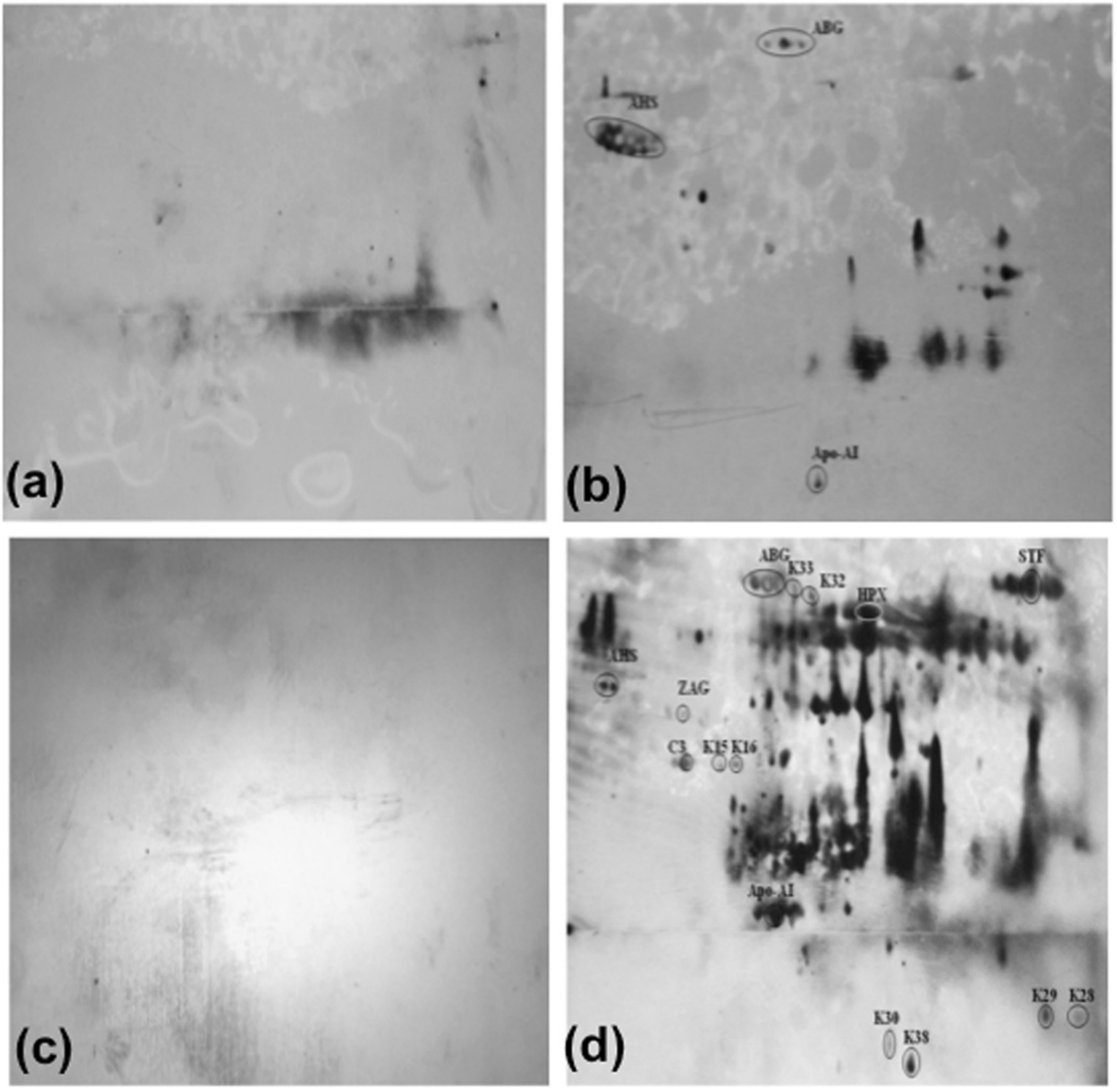
**Western blot results following 2-DE. (a)** normal pooled sera probed with normal pooled sera, **(b)** normal pooled sera probed with *P. knowlesi* pooled sera, **(c)** *P. knowlesi* pooled sera probed with normal pooled sera, **(d)** *P. knowlesi* pooled sera probed with *P. knowlesi* pooled sera, Unfractionated, pooled serum samples from patients and normal controls were subjected to 2-DE, transferred onto nitrocellulose membranes, and probed with pooled sera (as primary antibody) followed by monoclonal anti-human IgM-HRP (as secondary antibody).

**Figure 3:**
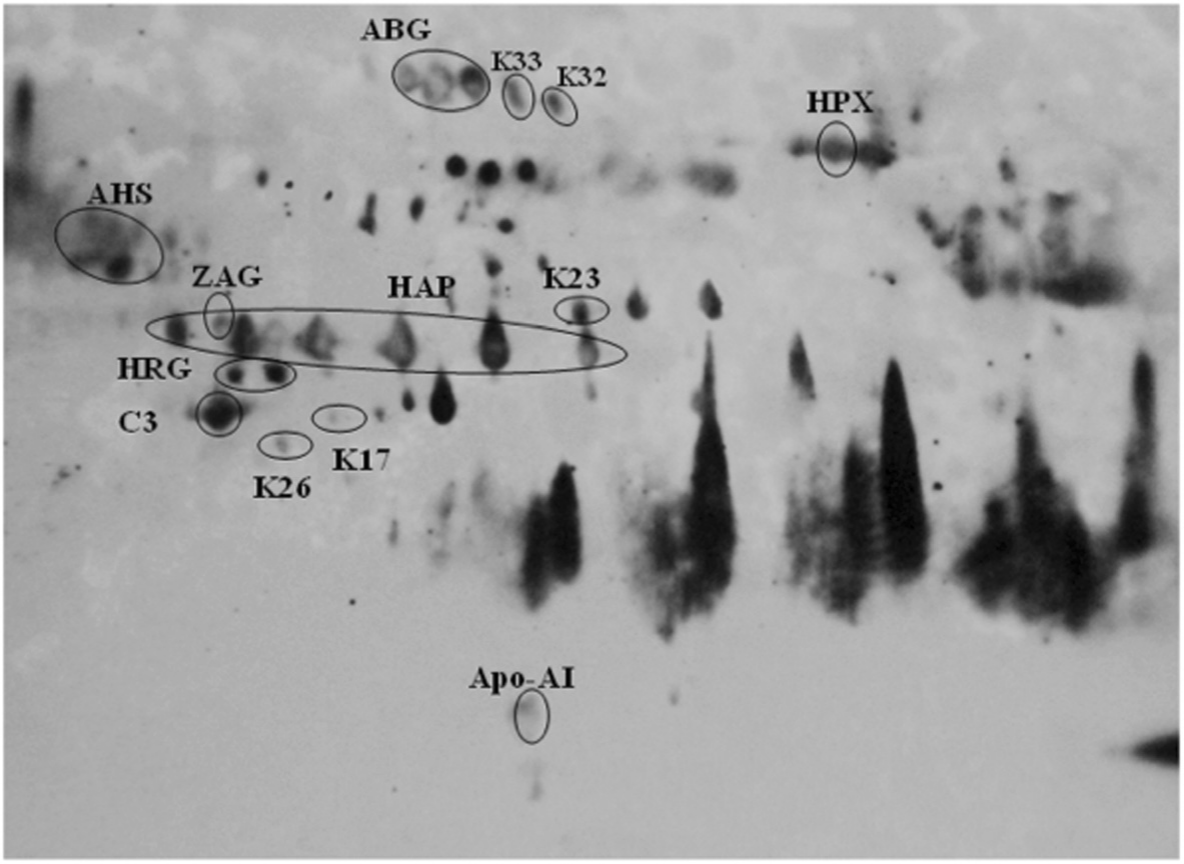
**Western blot of** ***P. knowlesi*** **pooled sera probed with** ***P. vivax*** **pooled sera.** Unfractionated, pooled serum samples from patient is subjected to 2-DE, transferred onto nitrocellulose membranes, and probed with pooled sera (as primary antibody) followed by monoclonal anti-human IgM-HRP (as secondary antibody).

In order to confirm the 2-DE image analysis and MS results, we performed immunoblotting with pooled patient sera (anti-*P. knowlesi* antibodies). Specifically, our immunoblotting analysis involved five distinct conditions to allow direct comparison of *P. knowlesi*-infected sera against normal sera and sera from *P. vivax*-infected patients. The following conditions were tested (categories a–e): (a) normal pooled sera probed with normal pooled sera, (b) normal pooled sera probed with *P. knowlesi* pooled sera, (c) *P. knowlesi* pooled sera probed with normal pooled sera, (d) *P. knowlesi* pooled sera probed with *P. knowlesi* pooled sera, (e) *P. knowlesi* pooled sera probed with *P. vivax* pooled sera are displayed in Figures 2 and 3. Immunoblotting of 2-DE membranes revealed significant variations in the control (Figure 2a, b) and patient (Figures 2c, d and 3) sera profiles. In Figure 2, only categories ‘a’ (negative control) and ‘c’ (positive control) failed to show immunogenic spots. However, the remaining categories (Figures 2b, d, and 3) revealed many immunogenic host-specific and malaria antigens. Strikingly, we could specifically distinguish differences when immunoblotting vivax malaria patients with sera from knowlesi-infected patients.

Immunogenic host-specific proteins are displayed in Table 2. Some of these malaria serum-reactive proteins could be detected in both non-infected and infected serum, including AHSG, A1BG, and Apo-AI (Figure 2b, d). However, ZAG1 and C3 appeared prominently for both individual patients in categories d and e. In addition, the A1BG signal was observed in categories d and e. Notably, HAP was the only protein that showed immunogenicity in category ‘e’ but not in category ‘d’. TF was detected in category ‘d’. HPX only appeared in the category ‘d’ immunoblot, whereas HRG was exclusively observed in category ‘e’.

With regard to malaria antigens, immunogenic spots for K15, K16, K28, K29, K30, and K38 were specific to *P. knowlesi*, as they could not be detected with antibodies from *P. vivax*-infected patients (Table 3 and Figure 3). However, only K15, K28, K30, and K38 were considered to be significant (i.e., search score of > 30). These four novel antigens were observed in 6 (66.7%), 5 (55.6%), 8 (95.1%), and 9 (100%) of the stained 2-DE gels, showing an average vol% of 0.00638, 0.00589, 0.04782, and 0.08956 (K15, K28, K30 and K38, respectively).

### Functional enrichment and protein interaction analysis

In order to extend the above results, we employed DAVID v6.7 (http://david.abcc.ncifcrf.gov/) to perform functional ontology enrichment analyses (i.e., biological processes, molecular functions, cellular components, and pathways) for 11 significantly identified proteins (Table 6). This functional analysis revealed that AHSG, C3, and histidine-rich glycoprotein precursor (HRG) were involved in regulating responses to external stimuli, whereas HAP, HPX and TF participated in iron homeostasis. In addition, molecular function analysis showed that AHSG, C3 and HRG play a role in endopeptidase and peptidase inhibitor activity. Also, the majority of the proteins were located in the extracellular region. However, cellular components analysis revealed that Apo-A1, fibrinogen γ chain (FGG), HRG, and TF could be found in secretory granules and membrane-bound vesicles. In addition, KEGG pathway analysis indicated significant participation of C3 and FGG in complement and coagulation cascades pathways (p = 0.026957).

**Table 6:** **Functional enrichment classification of identified host-specific proteins using DAVID bioinformatics tool**

		**Enrichment score** ^**+**^	**Protein count (%)**	**Protein ID**	**P value**
Biological process	Regulation of response to external stimulus	2.3	3 (27.27)	AHSG, C3, HRG	4.69e-3
	Cellular iron ion homeostasis	2.13	3 (27.27)	HAP, HPX, TF	1.81e-4
	Iron ion homeostasis	2.13	3 (27.27)	HAP, HPX, TF	2.45e-4
	Cellular di-, tri-valent inorganic cation homeostasis	2.13	3 (27.27)	HAP, HPX, TF	9.34e.3
	Di-, tri-valent inorganic cation homeostasis	2.13	3 (27.27)	HAP, HPX, TF	1.03e-2
	Cellular cation homeostasis	2.13	3 (27.27)	HAP, HPX,TF	1.16e-2
Molecular function	Endopeptidase inhibitor activity	2.3	3 (27.27)	AHSG,C3, HRG	3.32e-3
	Peptidase inhibitor activity	2.3	3 (27.27)	AHSG, C3, HRG	3.69e-3
	Enzyme inhibitor activity	2.3	3 (27.27)	AHSG, C3, HRG	1.11e-2
Cellular component	Extracellular region	8.11	11 (100)	A1BG, AHSG, APOA1, AZGP1, C3, FGG, HAP, HPX, HRG, LRG1, TF	2.66e-11
	Secretory granule	2.31	4 (36.36)	APOA1, FGG, HRG, TF	3.06e-4
	Cytoplasmic membrane-bounded vesicle	2.31	4 (36.36)	APOA1, FGG, HRG, TF	7.58e-3
	Membrane-bounded vesicle	2.31	4 (36.36)	APOA1, FGG, HRG, TF	8.29e-3
	Cytoplasmic vesicle	2.31	4 (36.36)	APOA1, FGG, HRG, TF	1.16e-2

^+^The classification stringency was set to high.

**Figure 4:**
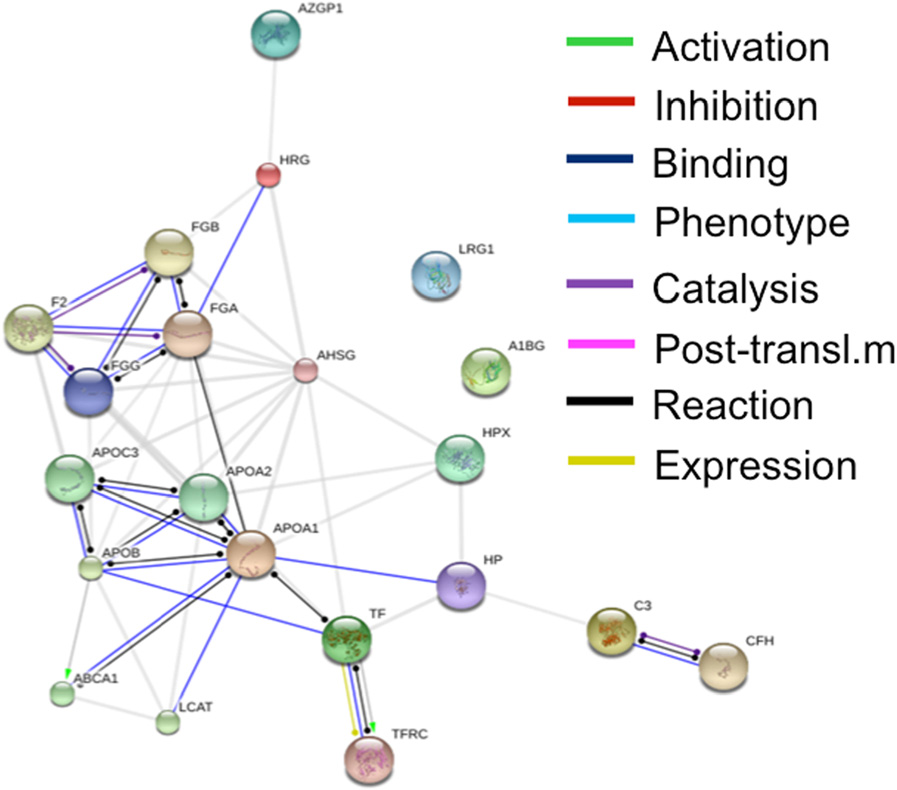
**Interaction networks of identified host specific proteins using STRING v9.1.** STRING database is a curated knowledge database that relies on various evidences from high-throughput proteomic, genomic and co-expression studies. The predicted functions of proteins are shown.

The STRING database (http://string-db.org/) is a curetted knowledge database that relies on evidence from high-throughput proteomic, genomic, and co-expression studies [19]. We performed protein interaction analysis using this database, which revealed an interaction network involving the identified host-specific proteins (AHSG, TF, C3, HPX, ZAG1, HAP, Apo-A1, HRG, A1BG, FGG and LRG1) (Figure 4). Indeed, most of the host-specific proteins display an established association with the Apo-A1-dominated network. Indeed, 10 protein partners (ABCA1, APOA2, APOB, APOC3, CFH, F2, FGA, FGB, LCAT, and TFRC) were predicted to be part of this functional interaction.

## Discussion

The global impact of malaria has sparked interest in developing effective diagnostic strategies, which are not only essential for resource-limited areas where malaria is a substantial societal burden, but also for developed countries [18,20]. In theory, the detection of malaria parasites or antigens in patient blood should be simple. However, diagnostic efficacy is subject to many important variables, including the various stages of erythrocytic schizogony, species endemicity, interrelationship between levels of transmission, population movement, parasitemia, immunity, and species-specific symptoms [11]. Among the *Plasmodium* species, detection of *P. knowlesi* is the most challenging due to morphological similarities and physical co-localization of genetic loci on the same chromosome within an individual or species. In this respect, Carlton et al. was the first to generate and compare a synteny map of *P. falciparum*, *P. vivax, P. knowlesi,* and the rodent malaria parasites (*P. yoelii, P. berghei, and P. chabaudi*) [19]. Overall, high synteny was observed between *P. vivax* and *P. knowlesi* chromosomes, with the exception of microsyntenic breaks in species-specific genes (e.g., *P. knowlesi kir* and *SICAvar*) [21]. These breaks were recognized as foci involved in the evolution of host–parasite interaction genes [22].

Proteomic analysis by 2-DE has been widely used to identify specific bacterial or viral antigens during vaccine and/or serodiagnostic test development [23]. Nevertheless, there have been few proteomic studies focused at elucidating pathogenic mechanisms or identifying potential diagnostic markers in malaria [24-26]. In the present study, we have analysed serum samples from infected individuals to identify and characterize novel markers of malaria infection using 2-DE coupled with immunoblotting techniques and mass spectrometry analysis. It is known that the immune response is relative with the presence of parasite density. Therefore, the immunoproteomics approach used in this study can be applied to identify antigens targeted by the immune system that response to infection. In addition, several studies have also reported on the protective role of IgM in the immune response during malaria infection [27,28]. Thus, both the host proteins and *P. knowlesi* antigens, represents the potential candidate biomarkers that could be used in the development of future diagnostic tools for *P. knowlesi*.

It has been suggested that invasion of erythrocytes by malaria parasites involves specific interactions between parasite receptors and erythrocyte ligands [29,30]. For this process, glycoproteins on the parasite receptors bind to hydrophobic peptides on the surface of human erythrocytes. Thus, hydrophobic peptides, which can be found in the form of sialic acid-rich regions, could make cells susceptible to infection by creating a negative charge at the surface [29]. Based on our findings, TF, HPX and HAP constitute very selective biomarkers for malaria infection. These three proteins are known as iron-binding glycoproteins which are involved in the regulation of iron homeostasis that plays a key role in the innate immune response [31].

Iron is essential for the development of malaria parasite. It is known that *Plasmodium* parasites synthesize their own TF receptors, which can become localized on the surface of infected cells [32-34]. The delivery of extracellular iron from TF to infected erythrocytes is the source of ferric ions for malaria parasites. The alteration of TF level may influence the balance between inhibiting and promoting the survival of malaria parasite [35]. Thus, it is possible that *P. knowlesi* and *P. vivax* directly secrete TF to gain access to iron through receptor-mediated endocytosis.

Notably, it was reported that iron deficiency induces TF receptor expression and doubles the number of HPX surface receptors (subsequently increasing HPX-mediated heme uptake *in vitro*) [36]. Under homeostasis, HPX can scavenge most of the free heme to form heme-HPX complex which prevent the onset of malaria [37]. Interestingly, some bacterial species, such as *Haemophilus influenzae*, *Campylobacter jejuni*, and *Yersinia pestis* [38-41], possess specialized iron acquisition systems for survival in hosts and are capable of heme uptake through heme–HPX complexes. Although the heme uptake activity is not parasite specific, it is possible that a similar survival system might be utilized by *Plasmodium* species. The expression of HPX has also been identified in several malaria studies, where this protein provides the support line of defence against haemoglobin-mediated oxidative damage during intravascular haemolysis [30,42].

Additionally, our results have demonstrated that HAP is downregulated in malaria patients. However, HAP was only antigenic in sera from *P. vivax*-infected individuals and not from *P. knowlesi*-infected patients. High HAP expression can reduce symptoms associated with malaria by causing toxicity to *Plasmodium* parasites [43] and by removing free haemoglobin (Hb) following *Plasmodium*-triggered haemolysis. In this regard, evidence has indicated that a higher peak of parasitemia and/or parasite burden was found in *P. berghei* (ANKA)- or *Plasmodium chabaudi*-infected mice compared to that of wild type [44]. In short, decreased expression of HAP could contribute to the life-threatening levels of parasitemia observed in *P. knowlesi*-infected patients*.*

## Conclusions

In summary, we have demonstrated the application of immunoproteomics approach to understand the immune response and identify potential candidate biomarkers for knowlesi malaria infection. Taken together, we have specifically identified TF, HPX and HAP as antigenic markers in *P. knowlesi*. A further investigation on the functional roles of the identified potential biomarkers in larger clinical samples will be valuable to enhance our current understanding of *P. knowlesi* and to develop effective diagnostic tools to detect knowlesi malaria.

## Abbreviations

DNA: Deoxyribonucleic acid
pLDH: *Plasmodium* lactate dehydrogenase
2-DE: Two-dimensional electrophoresis
SDS: Sodium dodecyl sulphate
CBB: Coomassie brilliant blue
MS: Mass spectrometry
CHCA: Cyano-4-hydroxy-cinamic acid
TFA: Trifluoroacetic acid
ACN: Acetonitrile
PMF: Peptide mass fingerprinting
TBST: Tris-buffered saline–Tween 20
HRP: Horseradish peroxidase
SD: Standard deviation
PCR: Polymerase chain reaction
AHSG: Alpha-2-HS glycoprotein
TF: Serotransferrin
C3: Complement C3c
HPX: Hemopexin
ZAG1: Zinc-2-alpha glycoprotein
Apo-A1: Apolipoprotein A1
HAP: Haptoglobin
A1BG: Alpha-1-B-glycoprotein
pI: Isoelectric point
Mr: Molecular mass
FGG: Fibrinogen γ chain
HO-1: Heme oxygenase-1
